# Development and Phenotypic Screening of an Ethyl Methane Sulfonate Mutant Population in Soybean

**DOI:** 10.3389/fpls.2018.00394

**Published:** 2018-03-29

**Authors:** Mary J. Espina, C. M. Sabbir Ahmed, Angelina Bernardini, Ekundayo Adeleke, Zeinab Yadegari, Prakash Arelli, Vince Pantalone, Ali Taheri

**Affiliations:** ^1^Department of Agricultural and Environmental Sciences, Tennessee State University, Nashville, TN, United States; ^2^United States Department of Agriculture, Agricultural Research Service, Jackson, TN, United States; ^3^Department of Plant Sciences, University of Tennessee, Knoxville, TN, United States

**Keywords:** EMS, soybean, mutation, tilling, late-maturity, phenotyping, seed compositions

## Abstract

Soybean is an important oil-producing crop in the Fabaceae family and there are increasing demands for soybean oil and other soybean products. Genetic improvement of soybean is needed to increase its production. In order to provide genetic diversity and resources for identifying important genes, a new ethyl methane sulfonate (EMS) mutagenized soybean population was generated using the newly released germplasm, JTN-5203 (maturity group V). Treatment of soybean seeds with 60 mM EMS concentration was found to be suitable for inducing mutation. A total of 1,820 M1 individuals were produced from 15,000 treated seeds. The resulting M2 population was planted in the field for phenotyping. After harvest, seed traits including total oil, protein, starch, moisture content, fatty acid and amino acid compositions were measured by NIR. Phenotypic variations observed in this population include changes in leaf morphology, plant architecture, seed compositions, and yield. Of most interest, we identified plants with increased amounts of total protein (50% vs. 41% for control) and plants with higher amounts of total oil (25% vs. 21.2% control). Similarly, we identified plants with increases in oleic acid content and decreases in linoleic acid and linolenic acid. This EMS mutant population will be used for further studies including screening for various traits such as amino acid pathways, allergens, phytic acids, and other important soybean agronomic traits. In addition, these mutant individuals will be evaluated in the next generation to assess the heritability. Beneficial traits from these mutants can be exploited for future soybean breeding programs. This germplasm can also be used for discovering novel mutant alleles and for functional gene expression analysis using reverse genetics tools such as TILLING.

## Introduction

Soybean (*Glycine max* L. Merrill) is an oil-producing legume crop in the Fabaceae family. Since the 1930s, soybean has been grown as an oilseed crop and contributed greatly to the recent United States economy (Wilson, 2008). It is the second most widely-grown crop in the United States next to corn (*Zea mays*), with a total area planted approximately 82.7 M acres in 2015 ($34.5 billion value). Soybean comprised 61% of the world oilseed production and is one of the major commercial crops worldwide (SoyStats, 2016). Aside from its oil content, soybean meal is a primary component of aquaculture and animal feeds (Wilson, 2008). In addition, the demand for soybean has increased as trends like biodiesel production is becoming popular in the market (Cao et al., 2005). With the increasing demand for soybean oil and other soybean products, it is imperative to increase its production. However, there are challenges that can affect the production of soybean including decreasing area for cultivation due to increasing population and climate change, abiotic stresses, and pest and disease problems (Hartman et al., 2011; Alexander et al., 2015; Iizumi and Ramankutty, 2015). To overcome these challenges in soybean production, and to meet the increasing global demands, soybean research communities are working to improve the genetic diversity that is available to plant breeders (Wilson, 2008; Patil et al., 2017).

Breeding of modern soybean varieties, which was focused on selection for modern traits such as higher yield, has created a genetic bottleneck for improvements in this crop (Valliyodan et al., 2016). These genetic bottlenecks resulted in loss of rare alleles and reduced the genetic diversity of soybean in the modern varieties (Hyten et al., 2006). One of the strategies that can be exploited to increase diversity in soybean genome is to introduce mutations. The reference soybean genome sequence (Schmutz et al., 2010) can be used to trace and identify mutations introduced to soybean. Plant mutation breeding involves harnessing the advantages of mutations to produce desirable variation for crop improvement (Pathirana, 2011). Gene mutagenesis changes nucleotide sequences, allowing production of novel alleles. There are four common mutagenesis methods (1) physical agents such as UV, X-ray radiation and fast neutron (FN), (2) chemical mutagens such as ethyl methane sulfonate (EMS), *N*-nitroso-*N*-methylurea (NMU), ethyl nitrosourea (ENU), 1,2:3,4-diepoxybutane (DEB), (3) biological agents such as T-DNA and transposons (Hancock et al., 2011), and (4) transgenic technologies such as CRISPR-Cas9, TALENs, gene knockdown using RNAi (Voytas and Gao, 2014; Lu et al., 2015).

Physical means of inducing mutagenesis include gamma radiation, X-rays, and neutrons. Fast neutron mutagenesis is conducted by treating the seeds with ionizing radiation and has been effective in plant mutation breeding. It creates large DNA fragment deletions resulting to loss of multiple candidate genes, serious damage to chromosomes, and large segmental duplications (Bolon et al., 2014; Oladosu et al., 2016). The deletions provide gene knock outs and phenotypic variations such as chlorophyll deficiency (Campbell et al., 2015), dwarfism (Hwang et al., 2014), hyper nodulation, and chimeras (Bolon et al., 2011).

Ethyl methane sulfonate is a chemical mutagen which is frequently used for seed mutation because it is effective and induces high frequency point mutations, some of which lead to a novel stop codon for different genes (Talebi et al., 2012; Chen et al., 2013). Aside from its effectiveness, EMS is also relatively easy to handle compared to other chemical mutagens such as nitroso compounds and can be detoxified via hydrolysis for disposal (Pathirana, 2011). Although several mutant populations have been developed earlier (Meksem et al., 2008; Bolon et al., 2011, 2014), the use of fast neutron and gamma radiation induces large-scale DNA deletion and inversion, which requires more study to narrow down the main gene involved in mutant phenotype. Point mutations produced by EMS can be detected using new technologies such as next generation sequencing and TILLING (Targeting Induced Local Lesion IN Genome). EMS mutagenized population can be analyzed using two approaches: (1) forward genetics, in which apparent phenotypes are characterized before the underlying gene is identified, and (2) reverse genetics, in which mutations in the genes of interest are detected first and later linked to a specific function or phenotype (Peters et al., 2003).

Soybean mutagenesis has been widely used to characterize loci controlling important functions, develop new varieties, discover new alleles, and screen for important agronomic traits (Cooper et al., 2008; Khan and Tyagi, 2013). For example, the disrupted version of *FAD2-1A gene* (omega-6 fatty acid desaturase; Glyma.03G144500) genes resulted in lower linoleic acid and higher oleic acid content, respectively (Meksem et al., 2008; Dierking and Bilyeu, 2009; Lakhssassi et al., 2017). Similarly, mutations in *RS2* (raffinose synthase; Glyma.03G137900) genes lead to an increase in sucrose levels and a decrease in both raffinose and stachyose oligosaccharide levels (Dierking and Bilyeu, 2009). Aside from above mentioned traits, soybean mutants have also been screened for phenotypic variations such as altered plant architectures, root phenotypes, and seeds colors (Bolon et al., 2011, 2014; Tsuda et al., 2015). Bolon et al. (2011) revealed different morphological phenotypes in FN mutant soybean plants including yellow pigmentation, curled leaves, early pod, hyper nodulation and non-nodulation, short trichome, chimeric and a short-petiole mutant with crinkled leaf (Bolon et al., 2011). Similarly, Tsuda et al. (2015) conducted consecutive EMS mutation in two generations and revealed large physiological and morphological phenotypes, resulting in a population with mutation rate of one mutation per 74 kb (Tsuda et al., 2015).

Although several mutant populations have been developed previously, most of them are from early maturity soybeans such as Williams 82 (MG III) which was the first reference genome (Schmutz et al., 2010), and only a few mutant populations were derived from late maturity cultivars including an older cultivar Forrest (Cooper et al., 2008; Lightfoot, 2008; Meksem et al., 2008). Modern day cultivars with higher yields and with other suitable traits need to be used in generating mutant population. To develop improved cultivars, there is a need to generate mutant population from late maturity groups, which are more adapted to lower latitudes. We choose to mutagenize the newly released germplasm, “JTN-5203” which is high yielding and has resistance to various diseases including soybean cyst nematode (SCN) (Arelli et al., 2015).

## Materials and Methods

### Plant Materials and EMS Treatment Optimization

All experiments were performed using soybean line JTN-5203 (Arelli et al., 2015). Based on previous reports (Meksem et al., 2008), three soaking times (12, 18, and 24 h) and six concentrations of EMS (0, 30, 60, 90, 120, and 150 mM) were tested to optimize the EMS concentrations. For each treatment, three replicates of 100 seeds were treated with EMS in 250 ml beaker at room temperature. Treated seeds were then washed three times with distilled water and sown immediately in 6 × 12 cell seed trays containing Fafard Professional Growing Mix No. 2. Germination rate was evaluated 21 days after sowing.

### Generating Mutant Population

To generate a M_1_ population, 15,000 bulk soybean seeds were treated in 60 mM EMS for 18 h. The majority of these seeds (10,000) were planted in the greenhouse while the remaining 5,000 were direct seeded in the field at 15.2 cm × 91.4 cm planting distance. Greenhouse grown seeds were transplanted individually to 6-inch pots and grown to maturity. M_2_ seeds were harvested from 1,820 surviving M_1_ plants, and about 12 M_2_ seeds/line were directly planted in the field in the following season (about 20,000 M_2_ plants) M_3_ seeds produced from 5,913 M_2_ population were stored for further characterization.

### Phenotyping

For each M_2_ family, 12 seeds were planted and survival rates were recorded at 3 weeks after planting. Lines with less than 50% germination were sown in the greenhouse and transplants were transferred into the field after 2 weeks. Survival rates were gathered 2 weeks after replanting. Visual phenotypic variation of growth behavior, leaf morphology, and branching was recorded and photos were taken for documentation in comparison to the wild-type, which is analogous to the depiction of FN mutants in SoyBase^1^.

### DNA Sample Collection and Extraction

Leaf samples were collected from 6,400 individual M_2_ plants that were tagged with barcoded labels. Freshly collected leaves were transported on ice and stored immediately in -80°C freezer. Frozen leaf samples were lyophilized using FreezeZone 6 Liter Console Freeze Dryer System (Catalog No. 7753524, Labconco, Kansas City, MO, United States). High-throughput tissue grinding were done in a 96-well plate (VWR Cat. No. 89005-562) format with stainless steel beads (one 3 mm per well) using TissueLyser System (QIAGEN, Valencia, CA, United States) as previously described by Meksem et al. (2008). DNA were extracted following modified CTAB method in a 96-well form (Perez et al., 2012), quantified using Synergy H1 Hybrid Reader (Biotek Instruments, Inc., Winooski, VT, United States), standardized, pooled and stored at -80°C.

### Post-harvest Data Gathering

Individual plant seed weight was measured for each individual mutant and wild-type. Cleaned seeds were weighed and protein, oil, fatty acids, sugar, and amino acids were measured from 12 g of seeds by near-infrared (NIR) spectroscopy with Perten Instrument (Model DA 7250, Perkin-Elmer, Inc., Perten Instruments North America, Springfield, IL, United States).

### Statistical Analysis

The experimental design used for optimization of EMS was a Complete Block Design and data were analyzed using R software (R Core Team, 2012). Analysis of variance was done and means were separated by Tukey’s HSD. For data generated from M_2_ population, only frequency distribution and the variance around the average of wild-type control plants with exceptional trait values was performed because the data were gathered from individual plants which were not replicated.

## Results

### Optimization of EMS Treatment

**FIGURE 1:**
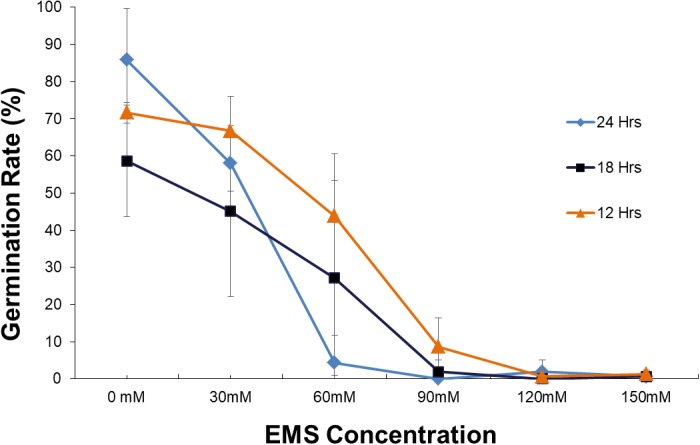
Plot of germination rate for various EMS concentrations (mM) and soaking time. Error bar represents the standard deviation of three replicates.

**FIGURE 2:**
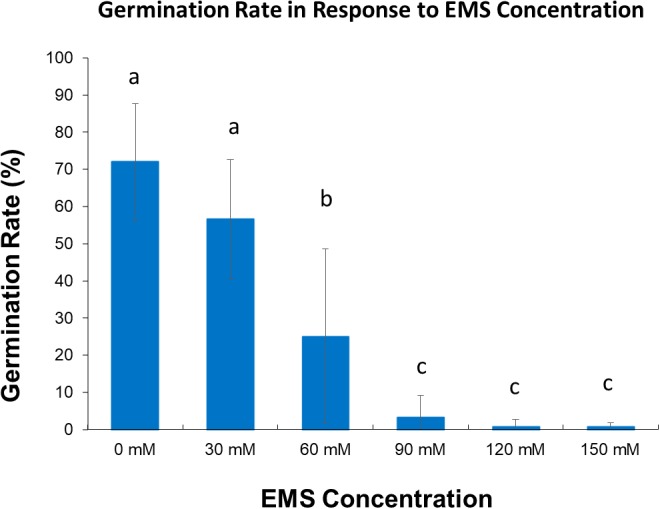
Chart of the effect of EMS concentration (mM) on germination rate for three soaking time (12, 18, and 24 h). Error bar represents the standard deviation of three replicates. Different letter indicates significant difference at α = 0.05.

Our first step was to evaluate the EMS treatment conditions for optimal mutagenesis. We tested a range of EMS concentrations and soaking times, both of which showed significant effects on germination rate. The germination rate decreases as the soaking time and the EMS concentration increases (**Figure 1**). Analysis by ANOVA showed that soaking time has effect on germination, separation of means by Tukey’s HSD showed the differences between soaking time are not significant. Although three soaking times 12, 18, and 24 h are not significantly different, 18 h was used in the experiment as it is within the range that Meksem et al. (2008) used which is 16–20 h. Statistical analysis of the effect of EMS concentrations on germination for three soaking time (12, 18, and 24 h) (**Figure 2**) showed that treatment with 30 mM is not significantly different from the control. However, treatment with 60 mM is significantly different from 0 to 30 mM and 90 to 150 mM (**Figure 2**) and resulted in about 50% survival rate.

Based on these results, we concluded that 60 mM (18 h) was the optimum concentration for our bulk EMS mutagenesis of soybean. While higher concentrations of mutagen would produce higher mutation frequency (Shah et al., 2015) 90 to 150 mM were found too detrimental to the genome resulting in very low germination. In contrast, if we use lower concentrations (30 mM) of EMS the survival rate of treated plants would be higher but the mutation rate will be low (Porch et al., 2009).

### Generation of Mutant Population

Using the optimized treatment protocol, a total of 1,820 M_2_ individuals were generated from 15,000 mutagenized seeds. There were two batches of mutagenesis, first batch with 10,000 mutagenized seeds generated 1,630 M_2_ individuals while second batch have 5,000 mutagenized seed generated only 190 M_2_ individuals. Batch 1 were planted in the greenhouse while Batch 2 were in the field. The difference in the survival/harvest was due to field condition where there was heavy rain for first couple of days after planting whereas in greenhouse plants were grown under optimal condition. The survival of germinated seedling was reduced due damaged cotyledon and to poor development of root and shoot. This type of tissue damage results from EMS mutagenesis and has also been observed pepper (Arisha et al., 2015) and cucumber (Shah et al., 2015) EMS treated seeds. In addition, some seedlings even grew until early vegetative stage but died before pod set.

### Phenotypic Variants in M_2_ Generation

**FIGURE 3:**
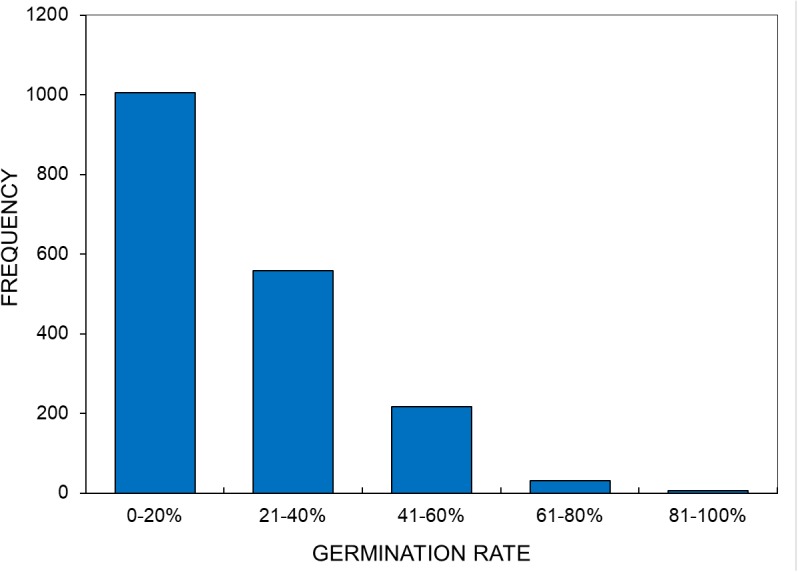
Frequency distribution of the germination percentages of M_2_ families (*n* = 1,820).

Previous studies have shown that EMS-induced mutation continues to affect germination and seedling survival of the M_2_ generation of peppers (Arisha et al., 2015), okra (Baghery et al., 2015), and soybean (Khan and Tyagi, 2013). This is likely the result of lethal mutations present in the M_1_ population becoming homozygous in the M_2_ generation. The frequency distribution of the germination rate (**Figure 3**) observed for various M_2_ lines which about 55% of the M_2_ lines have 0–20% germination, while about 2% of M_2_ lines have 61–100% germination. This result is similar to that observed for other experiments, suggesting that mutagenesis was sufficient enough to produce a high rate of mutations.

**FIGURE 4:**
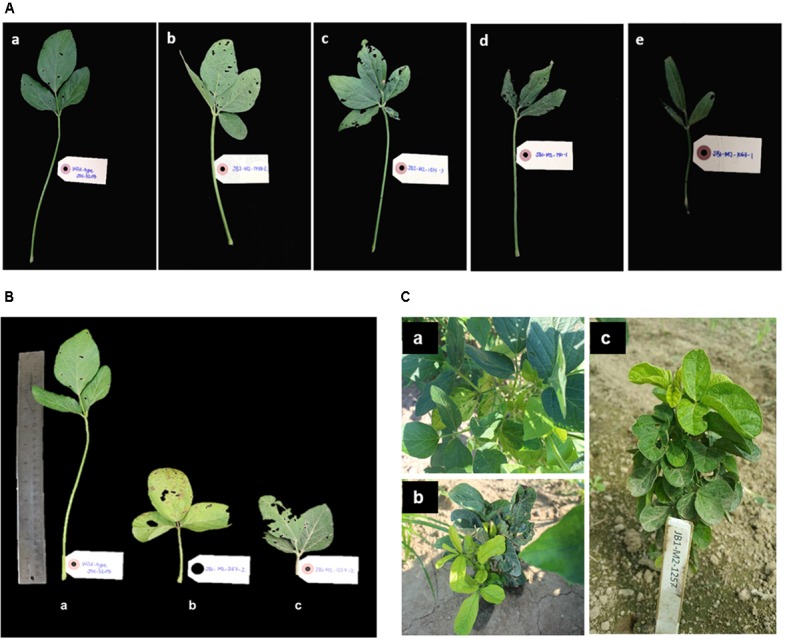
Phenotypic variation on leaf morphology observed in M_2_ plants. (**A**, top) Indicate phenotypic alterations observed in leaf morphology as compared to **(a)** wild-type JTN-5203, including **(b)** tetra-foliate, **(c)** penta-foliate **(d)** rough-textured leaf and **(e)** narrow leaf. (**B**, bottom left) Indicate variations observed in petiole length, with **(a)** wild-type JTN-5203 compared to **(b,c)** short-petiole mutant. (**C**, bottom right) Represent the changes in chlorophyll content including mutants with **(a)** chlorotic leaves, **(b)** chimeric and rough-textured leaves, and **(c)** compact plant with distinct yellow and green leaves.

Above ground visual phenotypic variation, including changes in leaf morphology, plant architecture, and changes in chlorophyll content, were also measured (**Table 1**). Statistical analysis was not performed in M_2_ generation as each plant is considered as one sample, which cannot be replicated. Statistical analysis of observed phenotypes will be conducted in the next generation planting with proper experimental design and replication, and to assess heritability of mutants. Some of the most striking phenotypic variations observed were altered leaf including, tetra-foliate (**Figure 4A-b**), penta-foliate (**Figure 4A-c**), rough texture (**Figure 4A-d**), and narrow leaf (**Figure 4A-e**) as compared to the wild-type JTN-5203 (**Figure 4A-a**). In some instances, tetra- and penta-foliate mutants were observed in just one or two leaves but did not penetrate the whole plant. Leaf phenotypes are important traits since they affect the leaf surface and the ability to perform photosynthesis. Another leaf phenotype documented is short-petiole leaf (**Figures 4B-a–c**). Abnormal chlorophyll phenotypes were also observed in several M_2_ plants including mutants with (**Figure 4C-a**) chlorotic leaves, (**Figure 4C-b**) chimeric and rough-textured leaves, and (**Figure 4C-c**) compact plant with distinct yellow and green leaves. Some of the mutants died, while other mutants that exhibit chimeric yellow leaves survived and produced some pods.

**Table 1:** Incidence of phenotypic variants observed from 6,400 M_2_ individuals.

Phenotypic variants	Incidence (%)	No. of mutants
***Leaf morphology***		
Tetrafoliate	0.23	15
Pentafoliate	0.14	9
Rough leaves	0.31	20
Narrow leaves	0.11	7
***Chlorophyll mutants***		
Cholorotic leaves	0.2	13
Chimeric leaves	0.17	11
Yellow plant	0.22	14
Stay green	0.34	22
***Plant architecture***		
Big plant	0.09	6
Bushy	7.47	478
Additional lateral branching	4.08	261
No lateral branching	0.34	22
Long internode	1.56	100
Dwarf/small plants	0.78	50
Thick stem	0.23	15
Short-petiole	0.03	2
***Reproductive***		
Dense pods	6.28	402
Sterile	7.63	488
No pod	3.23	207
Unfilled pod	4.09	262
Small seeds	0.33	21
***Other observed phenotypes***		
Lodging	2.92	190
Early maturity	1.45	93
Shattering^∗^	3.5	224

^∗^*Mutants observed with shattering potential might be due to late harvesting, but all shattering prone mutants observed were all accounted for*.

**FIGURE 5:**
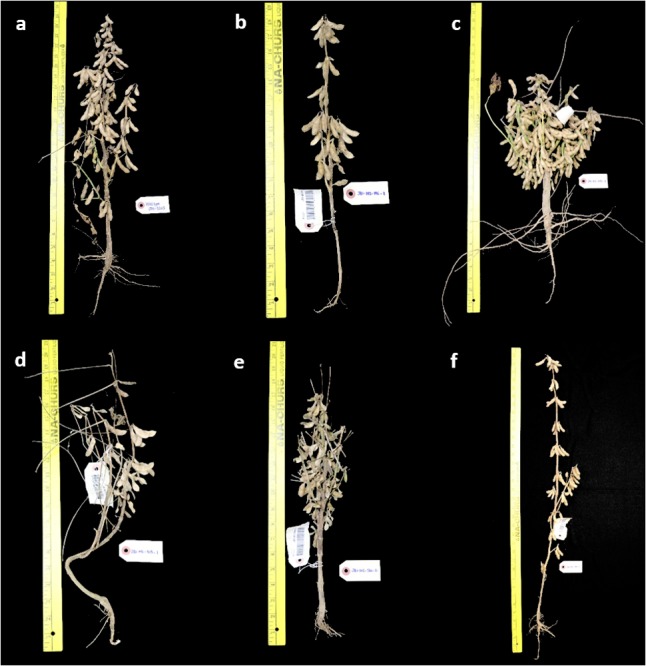
Phenotypic variations on plant architecture observed in M_2_ mutants. **(a)** Wild-type JTN-5203, **(b)** mutant without lateral branch, **(c)** bushy mutant with short internode, **(d)** unfilled pod mutant **(e)** shattering and early maturity mutant, and **(f)** increased in height.

**FIGURE 6:**
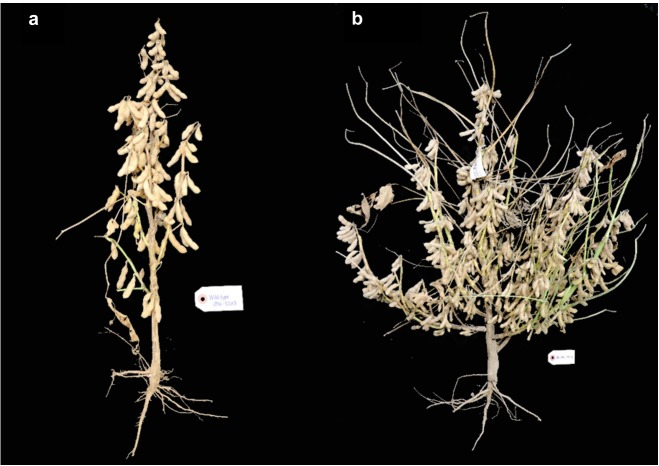
Comparison between wild-type **(a)** and an mutant **(b)** with additional lateral branching.

Several mutants with altered architecture and growth habit were also identified. Compared to wild-type (**Figure 5a**), these changes include, lack of lateral branching (**Figure 5b**), short internode and bushy type (**Figure 5c**), increased height (**Figure 5f**), unfilled pods (**Figure 5d**) and additional lateral branching (**Figure 6b** vs. **6a**). One mutant was called “bushy,” and was observed to have shorter internode, more branches and a thicker stem (**Figure 5c**). This may be a desirable agronomic trait if it proves to be resistant to lodging and produces additional pods per plant. However, there were also mutants that displayed more pods and shorter internodes. Similarly, mutants with reduced height (dwarf) but similar seed set may be useful for reducing cost of cultivation since fewer seeds would need to be planted per unit area to achieve sufficient number of pods (Hwang et al., 2014).

Other than leaf and architecture traits, other phenotypes such as sterility, lodging, and shattering (**Figure 5e**) were also observed. Some sterile plants did not develop pods at all, while some developing pods went unfilled. There were also mutants that were prone to lodging and shattering. These phenotypes are not favorable variations, but were recorded for future reference as they may provide clues about the genes required for agronomically important traits. For the mutants where interesting phenotypic variations were identified, they will be selected to be planted with replicates and appropriate experimental design in the next planting for further characterization and evaluation of heritability.

### Post-harvest Data

Because multiple seeds were planted for each M_2_ lines, we were able to harvest seeds from 5,913 M_2_ plants. Individual plant yield was measured and categorized based on the total seed weight per plant (**Table 2**). This analysis showed that about 78% of the plants had yields that are comparable to non-mutated control plants (average: ∼45 g, range = 32.1 to 55.7 g, *n* = 8). However, some mutants showed two to seven times higher yield than the controls. There were three mutant plants (JB1-M2-26-1, 251-1, and 224-1) that yielded more than 350 g. Since single plant yield is strongly affected by plant density, efforts are underway to determine the heritability of these high yielding lines.

**Table 2:** Individual plant yield of harvested M_3_ mutants^∗^.

Individual plant yield	M_3_ mutants		
	No. of Plants ±*SD*	Percentage
<50 grams	3306 ± 12.72	55.91
51–100 grams	1350 ± 14.72	22.83
101–150 grams	745 ± 13.98	12.60
151–200 grams	327 ± 14.22	5.53
201–250 grams	134 ± 13.00	2.27
251–300 grams	37 ± 15.54	0.63
301–350 grams	11 ± 9.68	0.19
>350 grams	3 ± 3.33	0.05

^∗^*Average individual plant yield for WT is 45 grams*.

**FIGURE 7:**
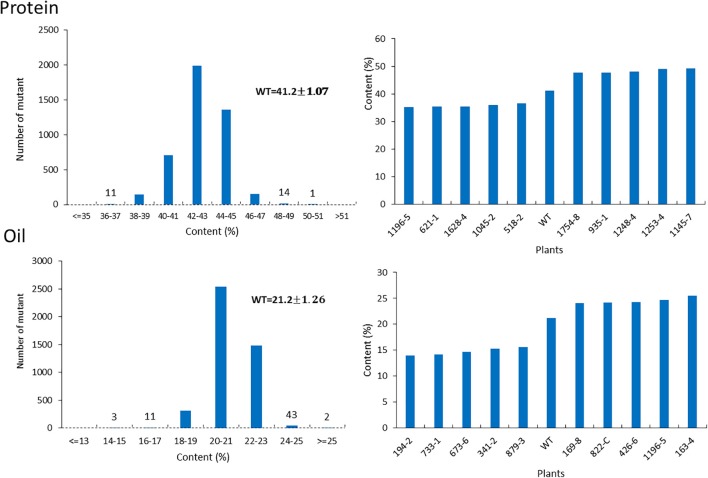
Protein and oil content in mutant seeds. Histograms **(left)** indicate variations in protein and oil content present in the population. Bold type indicates the average of protein and oil content in wild-type plants. Bar graphs **(right)** represent the protein and oil content values for average wild-type JTN-5203, top five and bottom five.

**FIGURE 8:**
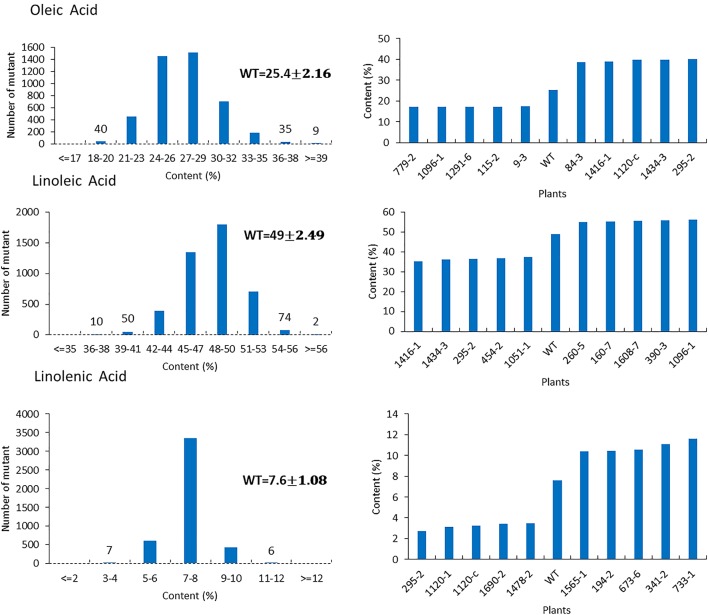
Fatty acids profile in mutant seeds. Histograms **(left)** indicate variations of oleic acid, linoleic acid, and linolenic acid oil content present in the population. Bold type indicates the average of oleic acid, linoleic acid and linolenic acid in wild-type plants. Bar graphs **(right)** represent the for average wild-type JTN-5203 top five mutants (and bottom five mutants for each fatty acid).

**FIGURE 9:**
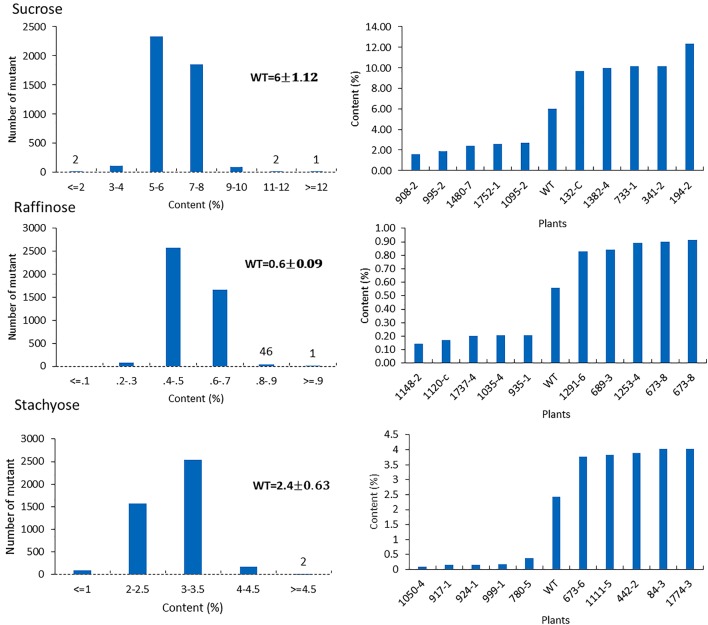
Sugar profile in mutant seeds. Histograms **(left)** indicate variations of sucrose, raffinose, and stachyose content present in the population. Bold type indicates the average of sucrose, raffinose, and stachyose in wild-type plants. Bar graphs **(right)** represent the for average wild-type JTN-5203 (top five mutants and bottom five mutants or each sugar).

**FIGURE 10:**
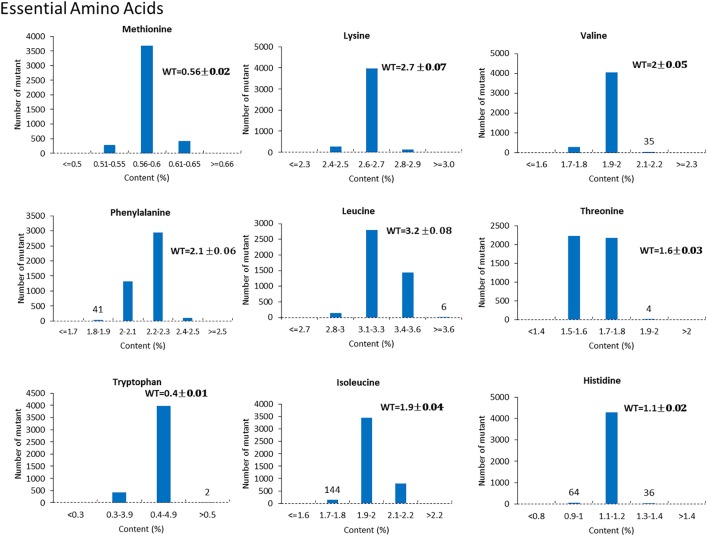
Variation of essential amino acid content in mutant seeds. Histograms indicate variations in methionine, lysine, valine, phenylalanine, leucine, threonine, tryptophan, isoleucine, and histidine content, respectively. Bold type indicates the average amino acid content in wild-type JTN-5203.

**FIGURE 11:**
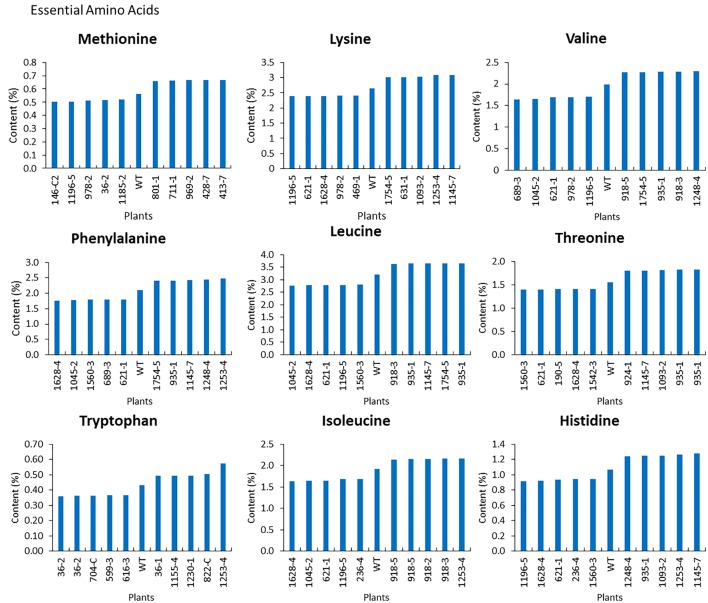
Essential amino acid profile of seeds from top and bottom five mutants. The average wild-type JTN-5203, top five and bottom five of methionine, lysine, valine, phenylalanine, leucine, threonine, tryptophan, isoleucine, and histidine are shown.

For the mutants that yielded at least 12 g of seeds, we performed NIR analysis (**Table 3**) to determine seed quality traits. The ranges of protein observed (35.2–49.15) was fairly diverse, forming a bell-shaped curve, suggesting that the mutagenesis has introduced additional variation (**Figure 7**). Surprisingly, total protein content was found to be higher in 80% of the lines. It is not clear if it is due to mutagenesis or if the control plants had lower protein content than normal due to location in the field or other variables. The range of total oil content (13.97–25.53) also suggests significant variation in the population, with 35% of the mutant population showing higher values than the control (**Figure 7**). The individual plants 1145-7 and 163-4 (**Figure 7**) have highest protein and oil content, respectively. Looking at the fatty acid profile, we identified nine mutants with increased oleic acid (39% compared to control average of 25%) (**Figure 8**). We also observed multiple mutants with decreased linoleic acid and linolenic acid (**Figure 8**). **Figure 9** shows the variation observe for sucrose, raffinose, and stachyose levels. Three mutants (733-1, 341-2, and 194-2) show almost double the sucrose content of controls. Similarly, a high number of mutants showed a decrease in raffinose and stachyose. We also found number of mutants with altered amino acid contents (**Figures 10**, **11**).

**Table 3:** Summary of NIR data for 4,389^∗^soybean mutants.

NIR variables	Mean	Minimum	Maximum
ADF	15.84	11.13	18.31
Alanine	1.8	1.56	2.01
Arginine	3.1	2.42	3.74
Ash	5.48	4.56	6.31
Aspartic	4.77	4	5.41
Cysteine	0.66	0.52	0.84
Fiber	5.8	3.34	7.34
Glutamic	7.4	5.9	8.54
Glycine	1.82	1.53	2.02
Histidine	1.1	0.91	1.28
Hydroxylysine	0.06	0.03	0.13
Hydroxyproline	0.08	0.06	0.11
Isoleucine	1.94	1.64	2.17
Lanthionine	0.13	0	0.22
Leucine	3.25	2.77	3.66
Linoleic	47.49	35.37	71.7
Linolenic	6.92	2.74	11.61
Lysine	2.74	2.38	3.09
Methionine	0.59	0.5	0.67
Moisture	8.79	6.32	12.79
Ndf	16.53	11.74	20.55
Oil	20.56	13.97	25.53
Oleic	26.63	16.76	39.98
Ornithine	0.04	0.02	0.12
Palmitic	11.77	6.28	14.84
Phenylalanine	2.14	1.76	2.49
Proline	2.12	1.75	2.46
Protein	42.31	35.2	49.15
Raffinose	0.48	0.14	0.92
Serine	1.91	1.61	2.29
Stachyose	2.13	0.12	4.04
Starch	6.08	0.53	11.46
Stearic	4.88	3.27	6.29
Sucrose	5.87	1.59	12.31
Taurine	0.03	0	0.16
Threonine	1.6	1.4	1.83
Tryptophan	0.43	0.36	0.57
Tyrosine	1.55	1.32	1.86
Valine	2.02	1.64	2.3

*Complete NIR data is provided in the Supplementary Data. ^∗^Although about 5,913 M_*3*_ mutants were harvested, NIR was only performed on the mutants which had at least 12 g of seeds*.

## Discussion

The success of mutation breeding program depends first and foremost on the effectiveness and efficiency of the mutagen used (Arisha et al., 2015). However, different mutagens have different effects based on the concentrations and the materials being treated. It is imperative to optimize the concentration of the mutagen before treating the bulk materials to ensure high mutation frequency and at the same time obtain enough viable seeds. High concentrations of mutagen are detrimental to plants, however, higher concentrations can also give higher mutation frequency (Shah et al., 2015). If the concentration of mutagen applied is too low, the survival rate of treated plants is higher although there is reduction in frequency of mutation (Porch et al., 2009). According to Cooper et al. (2008), an increase in mutation frequency can dramatically decrease the recovery of viable seeds. In case of EMS, an increase in concentration can significantly decreases the seed germination rate (Talebi et al., 2012).

A total of 6,400 individual M_2_ mutants were generated and DNA was collected for each plant. Several phenotypes were observed such as changes in leaf morphology, plant architecture, chlorophyll content, and germination rate. Leaf phenotypes such as narrow leaf, tetra-foliate, penta-foliate, and rough texture are important traits since they affect the leaf surface area. Leaf is the main site of photosynthesis, so the larger leaf surface area increases the photosynthesis rate as the leaf receives maximized sunlight for photosynthesis. Similar phenotypic changes have been observed with other mutagenesis programs. For example, the short-petiole phenotype was also observed in a common bean EMS mutant (Guner and Myers, 2001). The short-petiole phenotype is known to be controlled by single recessive gene *lps3* and is considered an important trait in increasing soybean yield by improving planting density and canopy profile (Jun et al., 2009). Also, EMS mutagenesis of peppers by Arisha et al., 2015, resulted in mutants with changes in leaf color indicating lesser chlorophyll content.

The characterization of mutant phenotypes can be useful in identifying the genes responsible for controlling plant growth and development. Of interest to our group are the changes in seed composition such as increase in total oil and proteins. There are also promising mutants that exhibited elevated levels of oleic acid and lower levels of linoleic and linolenic acid. Of interest, mutant 295-2 showed both higher oleic acid and lower linoleic and linolenic acid. These type of phenotypic alterations have been observed in previous studies and been shown to be associated with the *FAD2* gene in soybean (Meksem et al., 2008; Dierking and Bilyeu, 2009; Lakhssassi et al., 2017). We are also very interested in the mutants that demonstrate high yield potential with seven times higher single plant yield compared to the controls. Mutants showing interesting phenotypes such as additional lateral branching and dense pod set may also be able to contribute to higher yield potential. Maximizing yield is the first factor that farmers consider in selecting varieties to be planted. In evaluating yield potential of these mutants, it is will be important to evaluate the heritability of candidates and plant the mutants in multi-location yield trial that incorporate appropriate experimental design to determine if these mutations will consistently perform across different environments (Qin et al., 2015).

We have shown that the mutant library described in this study has been mutated sufficiently to produce observable phenotypic variations. Since the majority of the mutations present in the genome are recessive and are unlikely to produce obvious phenotypes, we anticipate that this population should be a valuable resource for functional genomics research in soybean. The use of PCR based strategy such as TILLING should allow identification of genes with interesting mutations. This can lead to an enhancement of the genetic variability that is useful in soybean breeding programs and in potential discovery of new alleles that may be valuable to the soybean industry. Identifying the underlying genes that control these interesting phenotypes will also be critical for understanding some of the functions of genes that are important for soybean improvement. For instance, a reverse genetics approach can be employed to identify novel alleles that are involved in biochemical pathways through finding gene homologs in the *Arabidopsis* genome. With TILLING, mutations in the genes will be mined from the mutant population, then individuals with the desired mutations will be subjected to phenotyping. Lines will eventually be selected and used for future breeding programs and markers can also be developed to aid in marker-assisted selection. In essence, trait discovery using this publicly available soybean mutant resource can be easily facilitated.

## Conclusion

We have used EMS to produce DNA mutations resulting in a mutant population with increased phenotypic variation. From this population, we have detected mutants with high oleic acid, oil, sucrose, protein and low linoleic and low linolenic acid contents that may be suitable for use in soybean breeding programs. In addition, further study is needed to fully understand and analyze the genetic and molecular changes underlying the phenotypic variability observed. After seed increase and confirmation of the phenotype in the next generations, mutants with interesting agronomic and value-added traits will be made available to Soybase for the scientific community access and to serve as public genetic resource for research and breeding programs. Finally, the overall goal of this project is to utilize EMS mutagenesis to develop and improve soybean germplasm and use this population as a reverse genetic tool in functional characterization of 50,000 predicted genes in soybean. Climate change leads to dryer growing seasons and emerging new pest and diseases and therefore soybean improvement and increasing its genetic diversity is necessary. Soybean is used in various industries such as animal feed, biodiesel, oil, and human food manufacturing and this mutant population is a valuable resource in screening for valuable traits in such industries as well.

## Author Contributions

AT, ME, CA, ZY, VP, and PA helped in writing the manuscript. PA provided the JTN-5203 soybean germplasm. ME, CA, AB, EA, ZY, and AT helped in planning and conducting the experiments.

## Conflict of Interest Statement

The authors declare that the research was conducted in the absence of any commercial or financial relationships that could be construed as a potential conflict of interest.

## Footnotes

1 https://www.soybase.org/mutants/index.php
